# Life history variation in Barents Sea fish: implications for sensitivity to fishing in a changing environment

**DOI:** 10.1002/ece3.1203

**Published:** 2014-09-02

**Authors:** Magnus A Wiedmann, Raul Primicerio, Andrey Dolgov, Camilla A M Ottesen, Michaela Aschan

**Affiliations:** 1 Norwegian College of Fishery Science, University of Tromsø9037, Tromsø, Norway; 2 Department of Marine and Arctic Biology, University of Tromsø9037, Tromsø, Norway; 3 Knipovich Polar Research Institute of Marine Fisheries and Oceanography6 Knipovich Street, 183038, Murmansk, Russian Federation

**Keywords:** Areal management, Barents Sea, biogeography, climate change, fast–slow continuum, fisheries, phylogeny

## Abstract

Under exploitation and environmental change, it is essential to assess the sensitivity and vulnerability of marine ecosystems to such stress. A species’ response to stress depends on its life history. Sensitivity to harvesting is related to the life history “fast–slow” continuum, where “slow” species (i.e., large, long lived, and late maturing) are expected to be more sensitive to fishing than “fast” ones. We analyze life history traits variation for all common fish species in the Barents Sea and rank fishes along fast–slow gradients obtained by ordination analyses. In addition, we integrate species’ fast–slow ranks with ecosystem survey data for the period 2004–2009, to assess life history variation at the community level in space and time. Arctic fishes were smaller, had shorter life spans, earlier maturation, larger offspring, and lower fecundity than boreal ones. Arctic fishes could thus be considered faster than the boreal species, even when body size was corrected for. Phylogenetically related species possessed similar life histories. Early in the study period, we found a strong spatial gradient, where members of fish assemblages in the southwestern Barents Sea displayed slower life histories than in the northeast. However, in later, warmer years, the gradient weakened caused by a northward movement of boreal species. As a consequence, the northeast experienced increasing proportions of slower fish species. This study is a step toward integrating life history traits in ecosystem-based areal management. On the basis of life history traits, we assess the fish sensitivity to fishing, at the species and community level. We show that climate warming promotes a borealization of fish assemblages in the northeast, associated with slower life histories in that area. The biology of Arctic species is still poorly known, and boreal species that now establish in the Arctic are fishery sensitive, which calls for cautious ecosystem management of these areas.

## Introduction

Life history traits determine, via their demographic implications, a species’ response to environmental stress such as harvesting (Sadovy 2001; Reynolds et al. 2005; Suding et al. 2008; Le Quesne and Jennings 2012). In fishes, the most studied among such so-called response traits are adult body size, size and age at maturity, longevity, fecundity, and offspring size (Jennings et al. 1998; Jeschke and Kokko 2009). These life history traits tend to covary due to the operation of correlational selection and trade-offs and are influenced by a species’ phylogeny and biogeography (Vila-Gispert et al. 2002).

A recurrent pattern of life history traits covariation arranges species along a “fast–slow” continuum (Promislow and Harvey 1990; Kozlowski 2006; Bielby et al. 2007). Typically, “fast” species can be characterized by being small and short lived, and by having small size and early age at maturation, whereas “slow” species are typified by the opposite properties (Kozlowski 2006). The fast–slow continuum does not imply r/K selection (Jeschke and Kokko 2009). Reproduction-related traits also covary, with fecundity and offspring size being negatively correlated due to an allocation trade-off (Stearns 1992).

Natural as well as anthropogenic factors may impose stress on ecosystems (Zacharias and Gregr 2005), and under environmental stress, particular life history strategies may be either beneficial or disadvantageous (Marshall 1953; Stearns 1992). For instance, slow fish species are thought to be demographically more sensitive to exploitation than species with faster life histories (Jennings et al. 1998; Denney et al. 2002; García et al. 2008). In this study, we define sensitivity as the degree to which fish species respond demographically to fishery-induced stress, as inferred from the species’ life history traits. Reproduction-related traits may also influence species’ sensitivity to fishing via their effect on fish recruitment, but the link depends on offspring survival which is notoriously difficult to predict, thereby weakening their value as sensitivity indicators (Sadovy 2001; Denney et al. 2002).

Although body size is recognized as a crucial factor determining a species’ sensitivity to fishing (Le Quesne and Jennings 2012; Dulvy et al. 2014), age and size at maturation appear to be important for such sensitivity as well (Jennings et al. 1999). Earlier maturation at smaller size results in shorter generation time, increased likelihood of surviving until maturation, and higher intrinsic rate of increase (Hutchings 2008; Stenseth and Dunlop 2009; Le Quesne and Jennings 2012). As such, short-lived species, such as the capelin (*Mallotus villosus*), often recover quickly from population declines (Hutchings 2000; Gjøsæter et al. 2009). Longevity, which tends to correlate with age at maturity, is associated with sensitivity as long-lived species often have poor recruitment in most years, relying on good recruitment in years with favorable environmental conditions. Therefore, long-lived species are likely to be especially sensitive to harvesting-induced age truncation (Dulvy et al. 2003). In addition, directed fishing may induce rapid evolution in life history traits, which involves increased fecundity, reduced annual growth, and earlier maturation at a smaller size (Yoneda and Wright 2004; Jørgensen et al. 2007; Swain et al. 2007; Enberg et al. 2012).

A ranking of species on the basis of their life history traits may allow for assessment of the species’ and communities’ sensitivity to fishing. The species composition of fish communities is influenced by rapid environmental change, thereby affecting the overall community sensitivity to fishing. Recent climate change has affected fish species distributions, inducing rapid changes in species composition (Last et al. 2011; Poloczanska et al. 2013; M. Fossheim et al.*,* unpubl. data). The climate-induced compositional changes are greater at higher latitudes due to the stronger exposure to climate warming near the Arctic, an area for which we have limited knowledge about the biology of the fish communities and their vulnerability to environmental stress (Gilg et al. 2012; Cristiansen and Reist 2013). In an area-based management context, it is important to map the life history variation for the regional species pool, and on that basis monitor change in overall community sensitivity in space and time.

**Figure 1:**
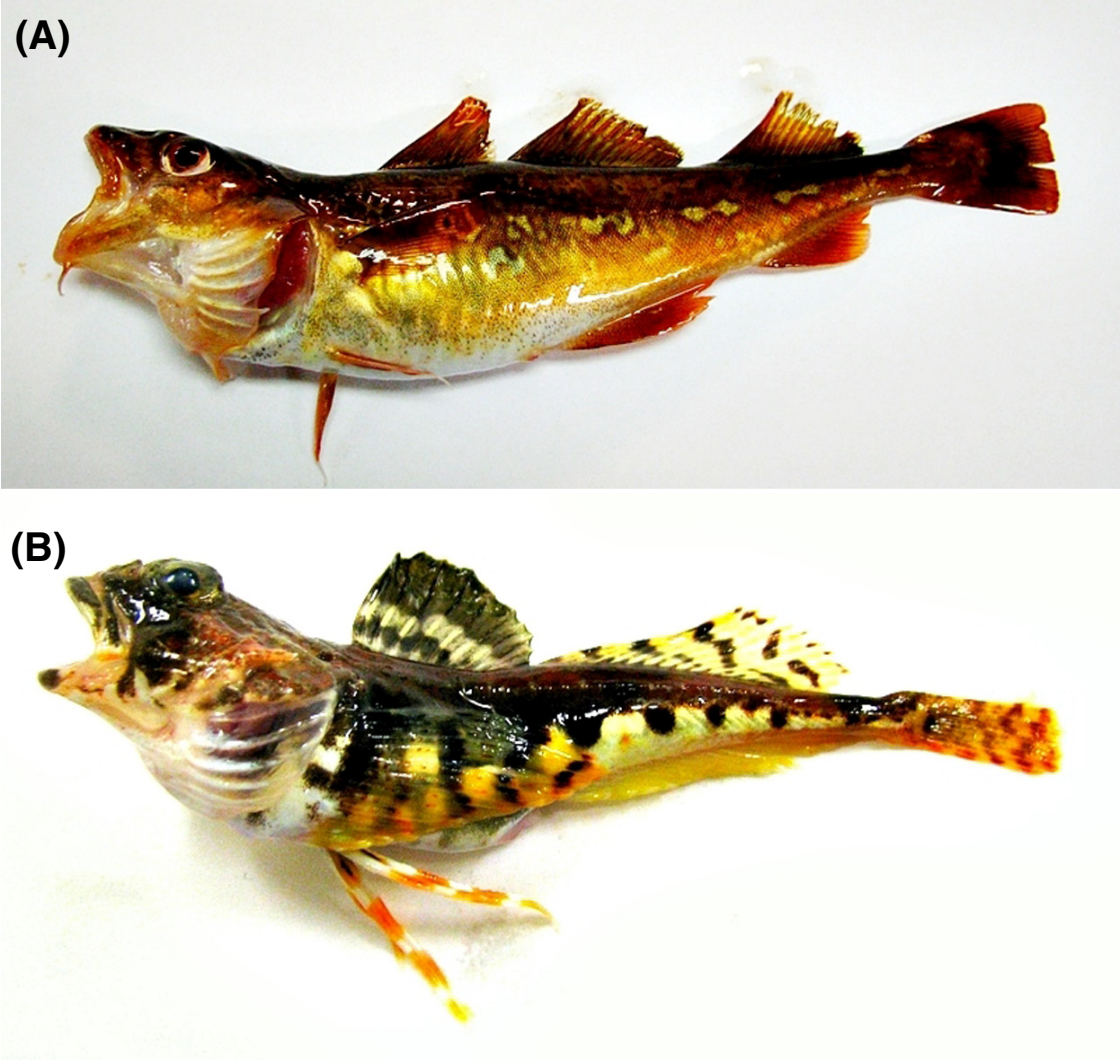
Fish species found in the Barents Sea. (A) A typical boreal species, the Atlantic cod (*Gadus morhua*). (B) A typical Arctic species, the Arctic staghorn sculpin (*Gymnocanthus tricuspis*).

In this study, we will map the life history characteristics of Barents Sea fish, which include boreal (Fig. 1A) and Arctic (Fig. 1B) species (Cristiansen and Reist 2013) and account for variation in phylogeny, biogeography, and habitat. Relative to boreal species, Arctic species are more closely related phylogenetically and subject to a strongly selective environment (Walsh 2008; Cristiansen and Reist 2013). Recent studies have revealed that the Barents Sea fish community can be characterized by distinct species assemblages, which are separated along gradients in space, temperature, and depth (Fossheim et al. 2006; Johannesen et al. 2012a). We extract species’ positions along gradients in life history strategies known to influence the species’ sensitivity to fishing. Furthermore, on the basis of ecosystem survey data from the Barents Sea, we assess such sensitivity at the community level for the years 2004–2009. In this period, the temperatures rose and the ice cover retreated in the Barents Sea (Johannesen et al. 2012b; Smedsrud et al. 2013), and as an effect, the fish community structure throughout the area has changed (Wiedmann et al. 2014; M. Fossheim et al.*,* unpubl. data). In particular, during the period 2004–2009, there was an increase in the number of species found in northern areas (Wiedmann et al. 2014), caused by a northward shift of boreal species such as cod (*Gadus morhua*) and long rough dab (*Hippoglossoides platessoides*) (Johannesen et al. 2012a; Johansen et al. 2013), which have different life histories than Arctic species. For the present paper, we had the following hypotheses:

1. We expected greater life history homogeneity among species members of Arctic assemblages due to their close phylogenetic relatedness and strongly selective environment as compared to more southern components of the Barents Sea fish community.
2. We hypothesized that fish assemblages in the Barents Sea would follow a latitudinal gradient, where the fishes residing in the northernmost areas display life history strategies most typical for high-latitude species: small size, high longevity, late age at maturity, low fecundity, and large eggs (Marshall 1953; Christiansen et al. 1998; La Mesa and Vacchi 2001; Peck et al. 2006; Hildebrandt et al. 2011).

## Material and Methods

### Study area

**Figure 2:**
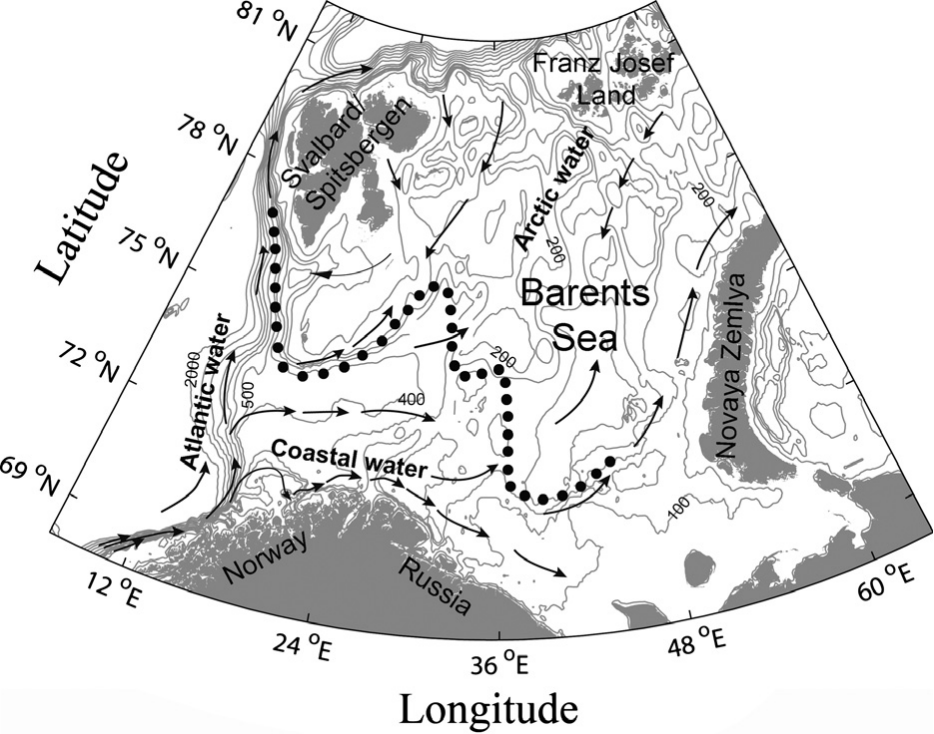
Map of the Barents Sea. Arrows indicate currents of the respective water masses. Dotted line indicates the mean position of the Polar Front, which separates Atlantic and Arctic water masses. The figure is based on Loeng (1991).

The Barents Sea is a shallow shelf sea in the Northeast Atlantic with an average depth of 230 m (Smedsrud et al. 2013). This area is delimited by the Norwegian and Russian coasts in the south, Novaya Zemlya in the east, the shelf break to the Atlantic Ocean in the west, and the shelf break to the Arctic Ocean in the north (Fig. 2). The climate in the Barents Sea is to a large degree governed by the inflow of Atlantic water (Smedsrud et al. 2013). Three distinct water masses with different temperatures (*T*) can be identified in the Barents Sea: Arctic water (*T* < 0°C), Atlantic water (*T* > 3°C), and mixed water (0°C < *T* < 3°C). The Polar Front separates Atlantic and Arctic water masses (Fig. 2). In later years, the proportion of mixed and Atlantic water masses have increased at the expense of the colder Arctic water masses (Johannesen et al. 2012b). There was a clear warming trend from the beginning of the period (2004) toward the later, warmer years 2007–2008, the latter 2 years being among the warmest years with the least ice cover and the longest ice-free seasons ever registered in the Arctic (Rodrigues 2009; Wadhams 2012). The proportion of Arctic water masses (water temperature <0°C) in the Barents Sea declined from 32% area coverage in 2004 to 22% in 2009, with proportions of 15–20% in the years 2006–2008 (Johannesen et al. 2012b). Conversely, the proportion of Atlantic and mixed water masses (water temperature >0°C) rose, from 68% in 2004 to 78% in 2009. Based on annual surveys carried out during fall, the average proportion of Arctic water masses in the Barents Sea was 35% in the 1970s, 39% in the 1980s, 35% in the 1990s, whereas the average for the 2000s was 26% (Johannesen et al. 2012b). In addition, a 4-degree summer warming of formerly ice-covered areas by 2050 is predicted (Smedsrud et al. 2013).

Of the ~220 fish species known to occur in the Barents Sea (A. Dolgov, unpubl. data), about 100 are commonly observed during annual ecosystem surveys (Wienerroither et al. 2011). The fish species include Arctic, arcto-boreal, and boreal species (Andriyashev and Chernova 1995). In addition to cod, several other commercially important species such as haddock (*Melanogrammus aeglefinus*), saithe (*Pollachius virens*), herring (*Clupea harengus*), and capelin (*Mallotus villosus*) reside in the Barents Sea (Olsen et al. 2010). During the recent period of rapid warming, boreal species such as cod, haddock, and beaked redfish (*Sebastes mentella*) have expanded their distribution northwards.

### Fish distributional data

Fishes were sampled in the period 2004–2009 during the Joint Norwegian–Russian ecosystem surveys organized by the Institute of Marine Research (IMR) and Knipovich Polar Research Institute of Marine Fisheries and Oceanography (PINRO) (Olsen et al. 2011). These surveys have been carried out annually (one survey each year) since 2003 in August–September, when the ice extent is at a minimum. The distance between sampling stations was 35 nautical miles (Johannesen et al. 2012a). In addition to fish, these surveys cover physical and chemical oceanography, plankton, benthos, sea mammals, seabirds, and contaminant levels (Michalsen et al. 2013). For demersal fish sampling, a Campelen 1800 shrimp trawl was towed for 15 min at 3 knots (0.75 nautical miles) at 50–500 m. In total, 98 fish species were caught at 1901 stations in 2004–2009. The number of trawled stations varied between years (from 319 in 2009 to 546 in 2005), but the total number of species (i.e., the species richness (SR)) did not vary much between years (from 66 in 2005 to 73 in 2008 and 2009). Species that were absent in more than 2 years or only caught off the chosen depth range were excluded from the analyses (*n* = 21). Two taxa (i.e., *Gymnelus* sp. and *Careproctus* sp.) were identified only to genus level due to uncertainties regarding species identification. Species richness per station varied between 1 and 21, and the final species list included 76 fish taxa (Table S9 in appendix S2, Supporting information). Bottom temperature and depth data were sampled at the stations using a conductivity, temperature, and depth (CTD) profiler.

### Life history traits data

We compiled a species*traits matrix for 76 Barents Sea fish species with the following 6 life history traits: longevity (years), age at maturity (years), maximum body size (cm), length at maturity (cm), fecundity (counts; number of offspring produced by a female per year), and offspring size (mm; egg diameter, capsule size in the case of skates, or size of newborn larvae in the case of ovoviviparity) (Table S1 in appendix S1, Supporting information). Trait information was compiled from literature, FishBase (http://www.fishbase.org; Froese and Pauly 2013), and from experts of Barents Sea fish (for citations, see Table S2 in appendix S1, Supporting information; for full reference list, see appendix S2). In cases where trait information was not available (*n* = 20, i.e., 4.4% of the total number of trait values), we estimated trait values based on knowledge about closely related species. The trait data were normalized via log_10_ transformation. Most trait values were compiled from studies of fish in the Barents Sea or environmentally comparable areas (see Table S3 in appendix S1, Supporting information, for reference to the locations from which the life history trait data were collected). Correlations among traits are shown in Table S4 (appendix S1, Supporting information).

### Explanatory variables

In order to explain life history patterns, we gathered information about biogeography, habitat preference, and diet (Table S5 in appendix S1, Supporting information). Also, phylogeny was accounted for by constructing a nested taxonomic matrix based on the following ranks: species, genus, family, order, class, and superclass (Table S6, appendix S1, Supporting information). The latter information was available at the World Register of Marine Species website (http://www.marinespecies.org/). The nested taxonomic matrix formed the basis for all phylogenetic analyses. In order to test for phylogenetic dependency in the life history traits gradients, we measured the phylogenetic signal (Blomberg et al. 2003). Also, we calculated phylogenetic diversity (PD) as the total branch length required to span a given set of taxa on the phylogenetic tree (Faith 1992). We further calculated phylogenetic dispersion (PDis) as the residuals from a linear model of PD (the response) as a function of SR (the predictor). Phylogenetic dispersion was used as a measure of phylogenetic diversity discounted for SR. Simply put, higher PDis at a sampling station means that the species found there are more distantly taxonomically related.

We classified the species according to their biogeographic affiliation (“Arctic,” “arcto-boreal,” or “boreal”), their habitat use (“demersal” or “pelagic”), and their feeding preferences (“benthivorous,” “planktivorous,” “piscivorous,” “bentho-piscivorous,” or “plankto-piscivorous”), based on literature (Andriyashev and Chernova 1995; Wienerroither et al. 2011) and expert knowledge.

### Ordination methods

**Figure 3:**
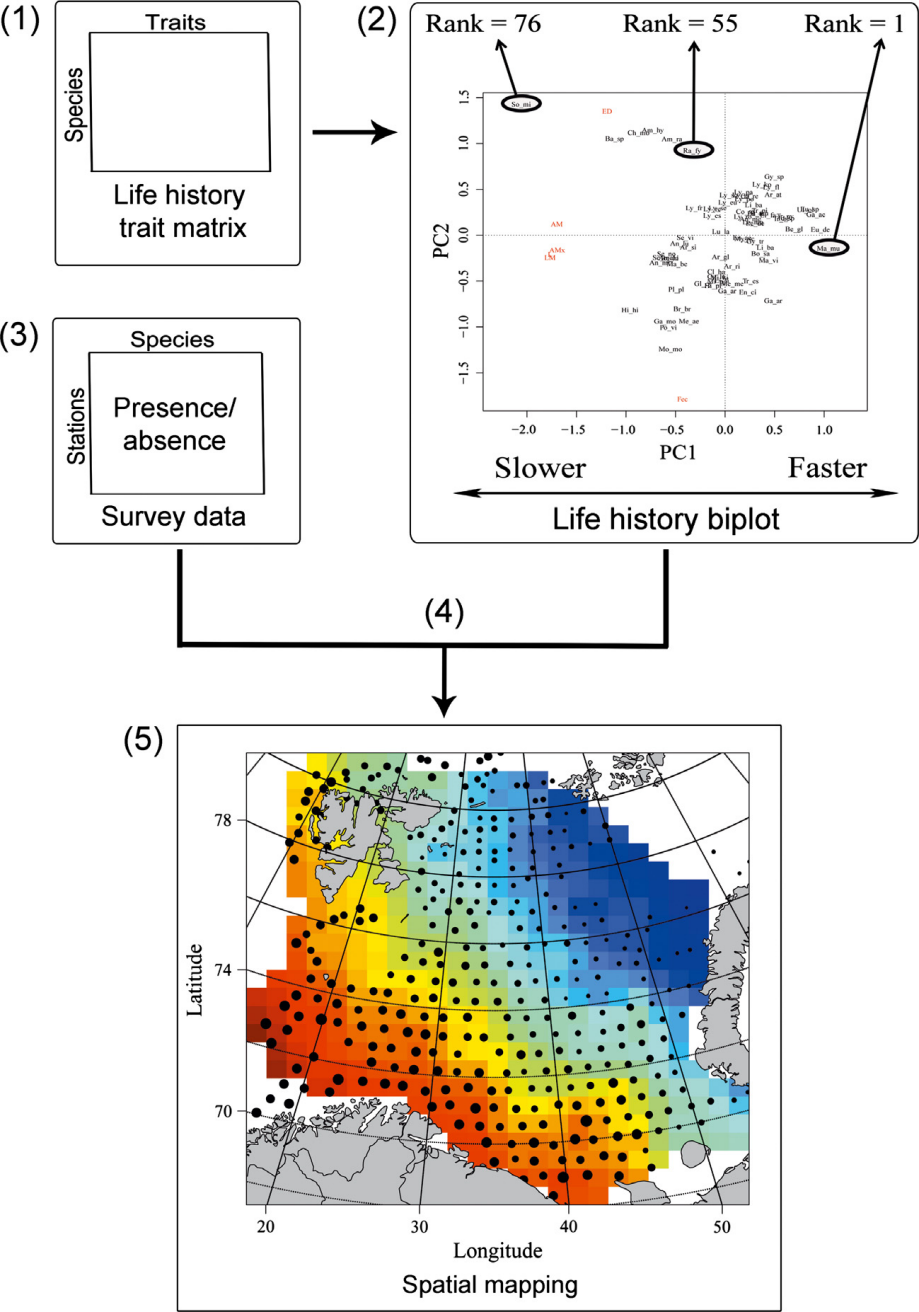
Procedure used to calculate and map fast–slow patterns in Barents Sea fish. (1) Construct life history matrix, (2) perform ordination to identify important patterns in the trait data, identify axes related to fast–slow continua, and extract the species’ ranks along these axes, (3) obtain spatiotemporal species data, (4) calculate the average rank of the species present at each station, and (5) map average values of species fast–slow ranks in time and space. Red colors represent assemblages composed of overall slow species (i.e., high values), whereas blue colors represent assemblages composed of overall fast species (i.e., low values).

We carried out two ordination analyses of the standardized trait data to identify main continua in life history traits covariation (Fig. 3). We first performed a principal component analysis (PCA). Secondly, as the covariation between life history traits can be confounded by body size (Jeschke and Kokko 2009), we performed a constrained ordination (redundancy analysis (RDA)) correcting for body size using this trait as a covariate (Legendre and Legendre 1998). For each ordination, we extracted the individual species’ positions (scores) along the main axes of life history variation associated, respectively, with the fast–slow continuum and the offspring size and number continuum. Species were ranked on the basis of their scores (from 1 to 76, where 76 was the number of species in the study).

In contrast to exact axis values, a simple ranking of species was chosen in order to account for trait uncertainty. Species with similar rank values will generally have similar trait values, and *vice versa*, but the ranking system does not carry explicit information about trait values.

### Fish community patterns in space and time

Estimated ranks along fast–slow life history gradients of single species were integrated with the ecosystem survey fish data (presence/absence). For each species assemblage at a station, we calculated and mapped the average species fast–slow ranks. For example, an average rank of 50 would be regarded as an assemblage composed of slow species, while an average rank of 30 would be regarded as assemblage composed of fast species. Assemblages mainly composed of slow species would typically be considered to be especially sensitive to fishing. Conversely, assemblages mainly composed of fast species would be considered to be less sensitive to fishing. For comparison with our estimates, we mapped mean estimates of species’ intrinsic vulnerability index available at www.fishbase.org (Cheung et al. 2005; Froese and Pauly 2013). The intrinsic vulnerability is derived from a fuzzy expert system approach applied to species’ life history and ecological traits. Its working principle resembles the fast–slow approach in that it recognizes that certain life history strategies (e.g., large body size and late maturation) increase a species’ susceptibility to suffer fisheries-induced extinction, disregarding other sources of mortality such as pollution (Cheung et al. 2005). We also mapped PDis to study the spatial variation in phylogenetic distance between species caught at sampling stations. Interpolation between stations was carried out using universal kriging (Cressie 1993).

We modeled variation in phylogenetic dispersion and average fast–slow ranks as functions of water temperature and depth using generalized linear models (GLM). Spatial autocorrelation was accounted for by including geographical coordinates and associated polynomial terms in the model.

We used the following R (R Core Team 2012) libraries: vegan (Oksanen et al. 2012) for multivariate analyses and to calculate phylogenetic diversity, picante (Kembel et al. 2010) to calculate phylogenetic correlation, gstat for spatial modeling (Pebesma and Wesseling 1998), maptools (Lewin-Koh and Bivand 2013) and fields (Furrer et al. 2012) for mapping.

## Results

### Life history variation and fast–slow continua

**Figure 4:**
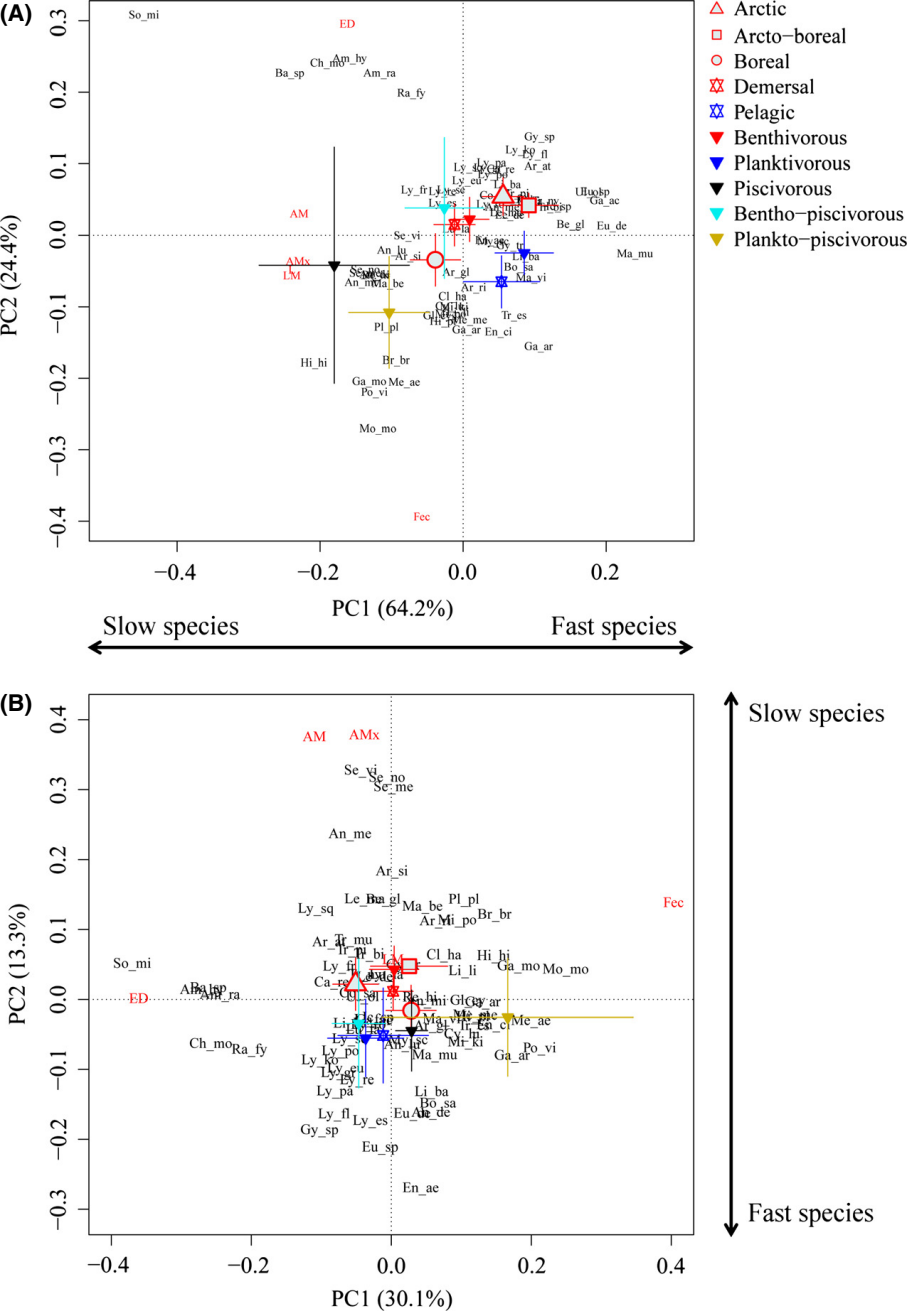
Biplots of life history variation in 76 Barents Sea fish species, based on (A) PCA, which was used to extract the FS 1 ranks and (B) RDA using body size as constraining variable, which was used to extract the FS 2 ranks. Trait abbreviations: ED = offspring size; Fec = fecundity; AM = age at maturity; AMx = maximum age; L = maximum body size; LM = length at maturity. Covariate centroids are shown as symbols with 95% confidence intervals.

Ordination biplots illustrated covariation in Barents Sea fish life history traits (Fig. 4). In the PCA (which was not corrected for body size), PC1 accounted for 64.2% of the variation, whereas PC2 accounted for 24.4% of the variation (Fig. 4A). The traits related to the fast–slow continuum (maximum length, length at maturity, longevity, and age at maturity) were strongly correlated, and all loaded strongest on the PC1. Therefore, we extracted the species’ positions along the PC1 axis, and we termed the resulting species ranking “Fast–Slow 1” (FS 1). In addition, we extracted the species’ positions along the PC2, which was governed by reproduction-related traits. We termed the resulting species ranking the “offspring size and number continuum.”

In the RDA, which accounted for body size, PC1 and PC2 accounted for 30.1% and 13.3% of the variation, respectively, whereas the constrained axis (RDA1) accounted for 48.6% of the variation. Here, PC1 showed a clear continuum from species with few, large offspring to species with many, small offspring, whereas PC2 captured the fast–slow continuum (Fig. 4B). Indeed, the PC2 from the RDA is interesting in a fast–slow context as this axis explicitly corrects for variations in body size. We therefore extracted the species’ positions along the PC2 axis, and we termed the resulting lists of species’ positions along this fast–slow gradient “Fast–Slow 2” (FS 2).

On average, the boreal species had higher fecundity, smaller offspring, and larger size as compared to the Arctic species (Fig. 4A,B). However, when correcting for body size (i.e., FS 2), the boreal species did not have significantly higher longevity than the Arctic species (Fig. 4B). The piscivorous and demersal species were largest and had the highest longevity (Fig. 4A), but the benthivores had higher average longevity than the piscivores when body size was corrected for (Fig. 4B).

**Figure 5:**
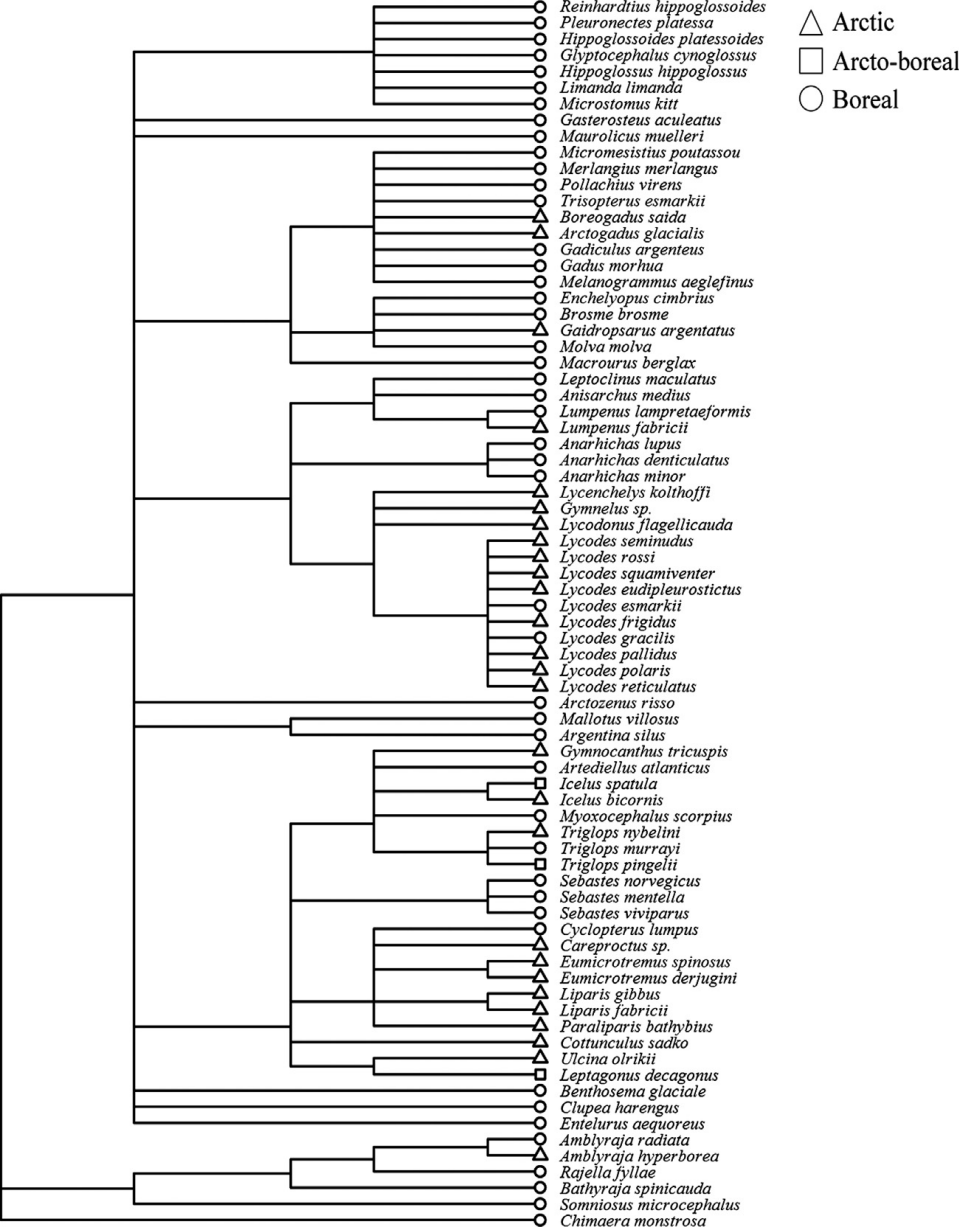
Phylogenetic dendrogram of 76 Barents Sea fish species. Tip symbol reflects the species’ biogeographic affiliation.

Closely taxonomically related species generally displayed similar biogeographic affiliation (Fig. 5). For instance, most eelpouts (*Lycodes* spp.) were of Arctic origin, whereas the wolffishes (*Anarhichas* spp.) were boreal.

**Figure 6:**
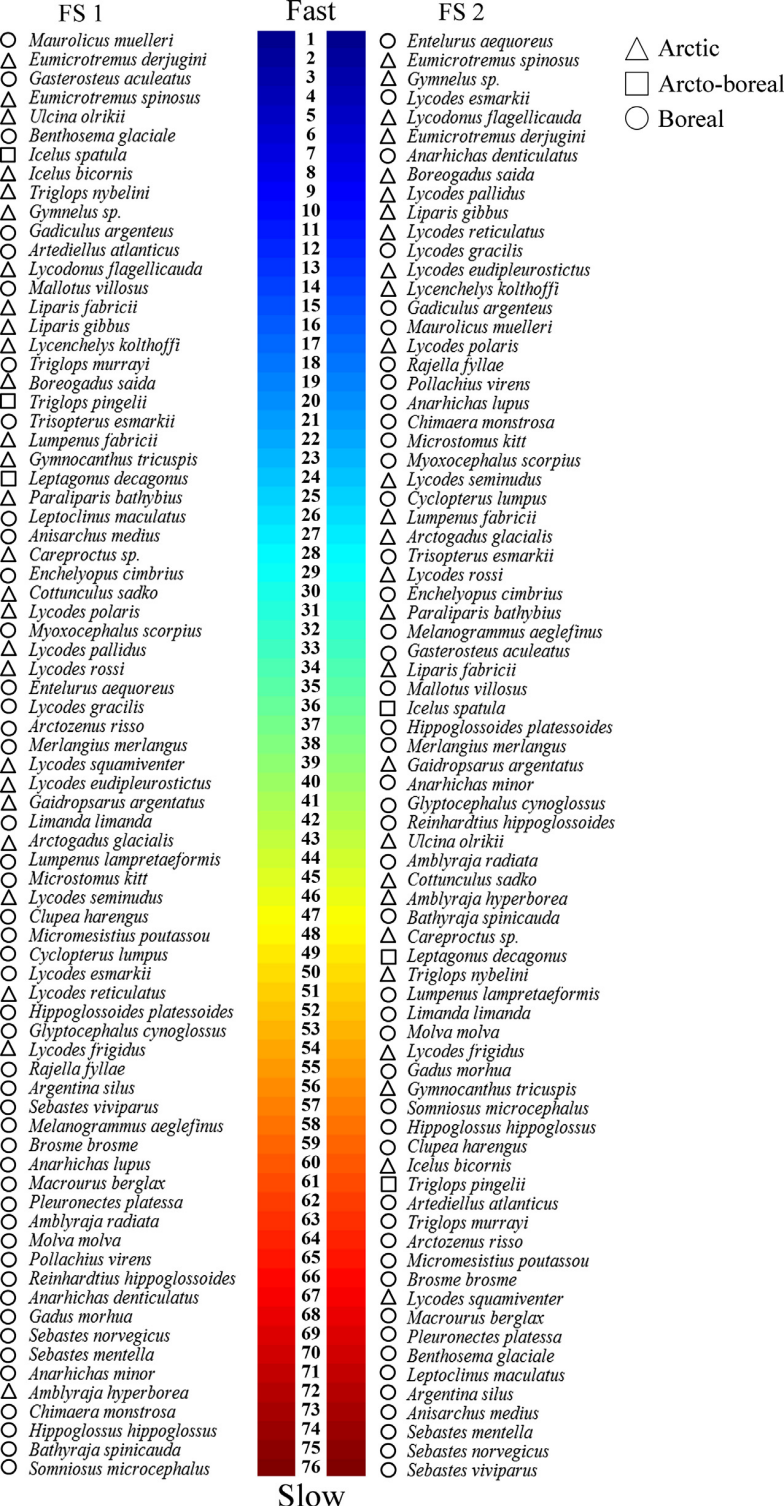
Ranking of Barents Sea fish species along life fast–slow axes. FS 1 (PC1 from a PCA) is driven by the body size, size at maturity, longevity, and age at maturity, whereas FS 2 (PC2 from an RDA) corrects for maximum body size.

The Arctic species (*n* = 27) showed a mean FS 1 rank of 27 and a mean FS 2 rank of 28, whereas the boreal species (*n* = 46) showed a mean FS 1 rank of 47 and a mean FS 2 rank of 44 (Fig. 6). The arcto-boreal species (*n* = 3) had a mean FS 1 rank of 16 and a mean FS 2 rank of 49. The redfish (*Sebastes* spp.) as well as the elasmobranchs and several of the species of order Gadiformes (e.g., the roughhead grenadier, *Macrourus berglax*) showed consistently slow life histories (Fig. 6). The eelpouts (*Lycodes* spp.) were generally faster. Some inconsistency was observed between the two FS ranks. For instance, the rabbit fish (*Chimaera monstrosa*) turned out to be slow according to FS 1 and faster according to FS 2. Most of the slow species were classified as boreal. The slowest Arctic species was the arctic skate (*Amblyraja hyperborea*), which was ranked as number 72 according to FS 1 and number 46 according to FS 2.

There were highly significant phylogenetic signals in both FS 1 (*P* = 0.001) and FS 2 (*P* = 0.002), implying that both approaches showed statistical dependency due to the species’ phylogenetic relationships. Also the offspring size and number gradient showed significant phylogenetic dependency (*P* = 0.001). All explanatory variables (diet, habitat, and biogeography) could explain the life history patterns shown in Fig. 4 (*P* < 0.05).

### Life history variation in space and time

**Figure 7:**
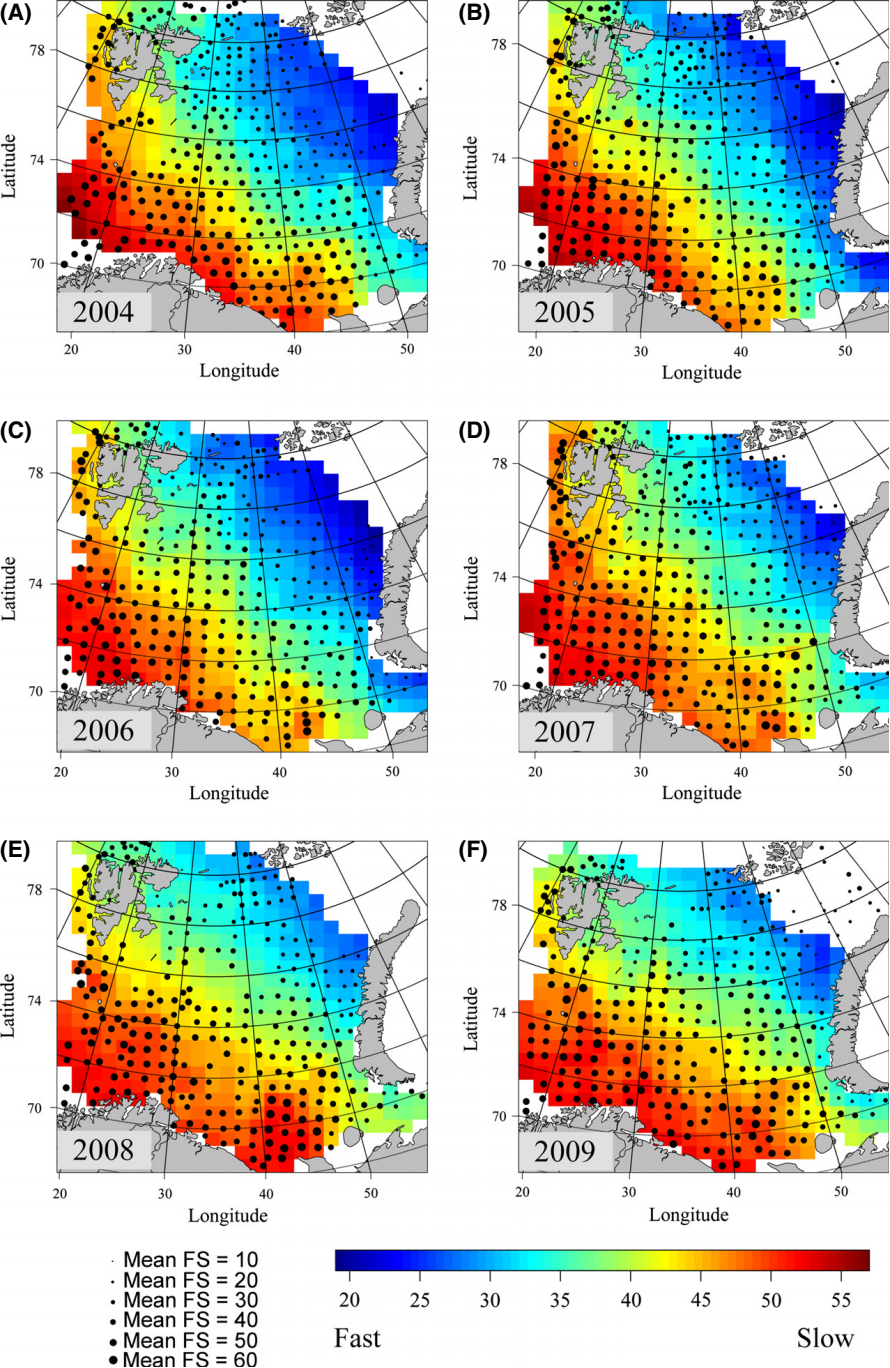
Mean ranking of life history speed of Barents Sea fish assemblages, 2004-2009, according to FS 1. Higher FS values (red) indicate assemblages consisting of overall slow species, whereas lower FS values (blue) indicate assemblages consisting of overall fast species. (A) 2004, (B) 2005, (C) 2006, (D) 2007, (E) 2008, (F) 2009.

**Figure 8:**
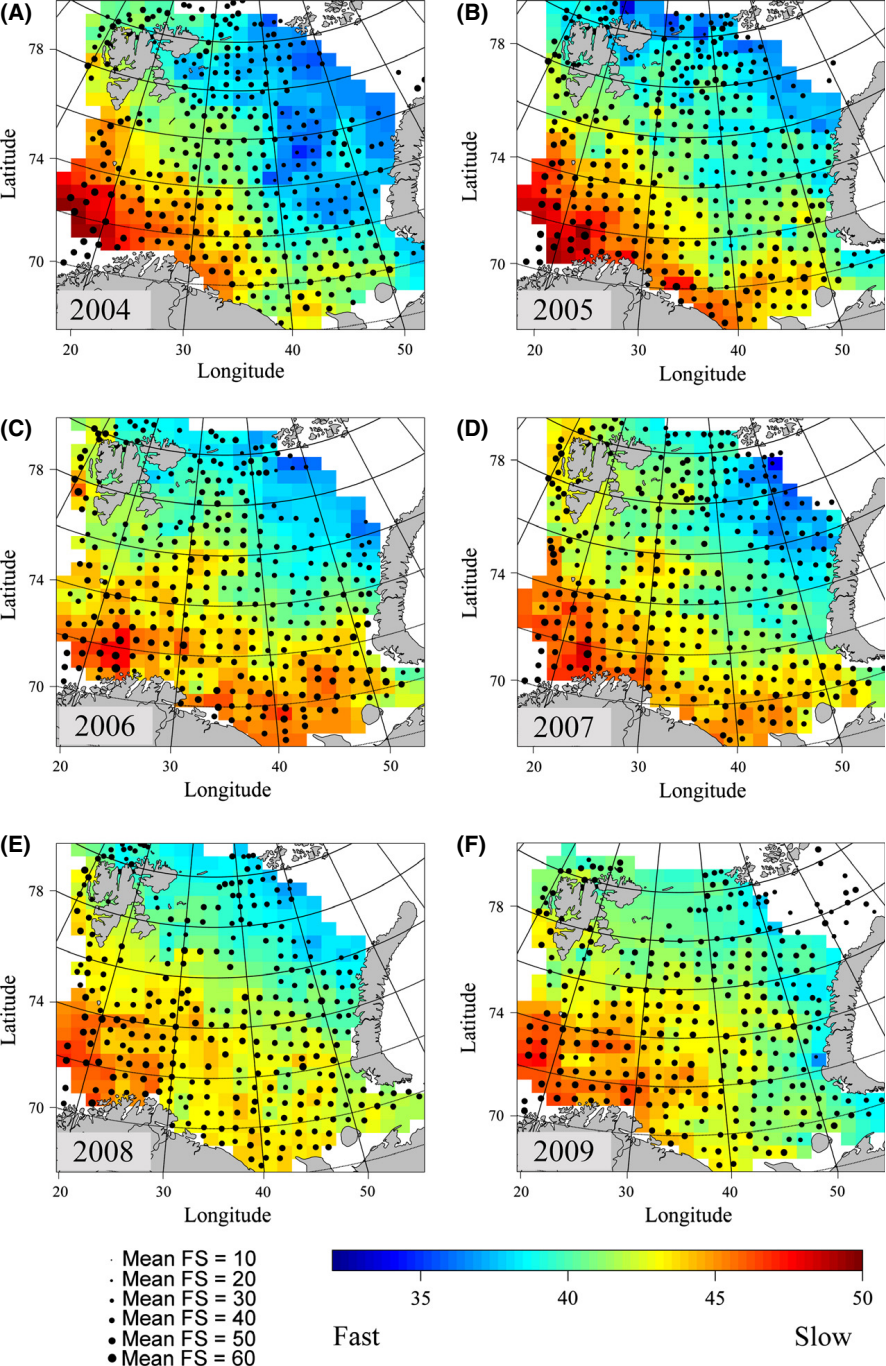
Mean ranking of life history speed of Barents Sea fish assemblages, 2004–2009, according to FS 2 (corrected for body size). Higher FS values (red) indicate assemblages consisting of overall slow species, whereas lower FS values (blue) indicate assemblages consisting of overall fast species. (A) 2004, (B) 2005, (C) 2006, (D) 2007, (E) 2008, (F) 2009.

Mapping average values of FS 1 and FS 2 at sampling stations, where FS 2 was corrected for variation in body size, showed similar patterns with respect to space and time (Figs 7 & 8). Both approaches showed a notable spatial gradient, with generally slow species in the southwest and faster species in the northeast. When ignoring variation between years, the FS 1 average values showed a significant positive correlation with temperature (estimated marginal effect of 2.26 increase in mean FS 1 value per one degree increase in temperature, standardized regression coefficient = 0.229, *P* < 0.001) and depth (estimated marginal effect of 1.49 increase in mean FS 1 value per 100 m increase in depth, standardized regression coefficient = 0.151, *P* < 0.001). FS 2 also showed significant correlations with temperature (estimated marginal effect of 0.45 in mean FS 2 value per one degree increase in temperature, standard regression coefficient = 0.088, *P* = 0.02) and depth (estimated marginal effect of 0.35 increase in mean FS 2 value per 100 m increase in depth, standardized regression coefficient = 0.067, *P* = 0.01). More detailed analyses of the northeastern part of the Barents Sea confirmed the stronger rise in average FS 1 and FS2 values there as compared to the Barents Sea as a whole (Fig. S7 in appendix S3, Supporting information). We also note that the assemblage average values for the offspring size and number gradient (i.e., species ranks resulting from the PC1 in the RDA) decreased from the southwest to the northeast, with fishes in the northeast producing larger, fewer offspring. Typically, average ranks along the offspring size and number gradient were above 50 in the south and below 40 in the northeast (Fig. S8 in appendix S3, Supporting information).

**Figure 9:**
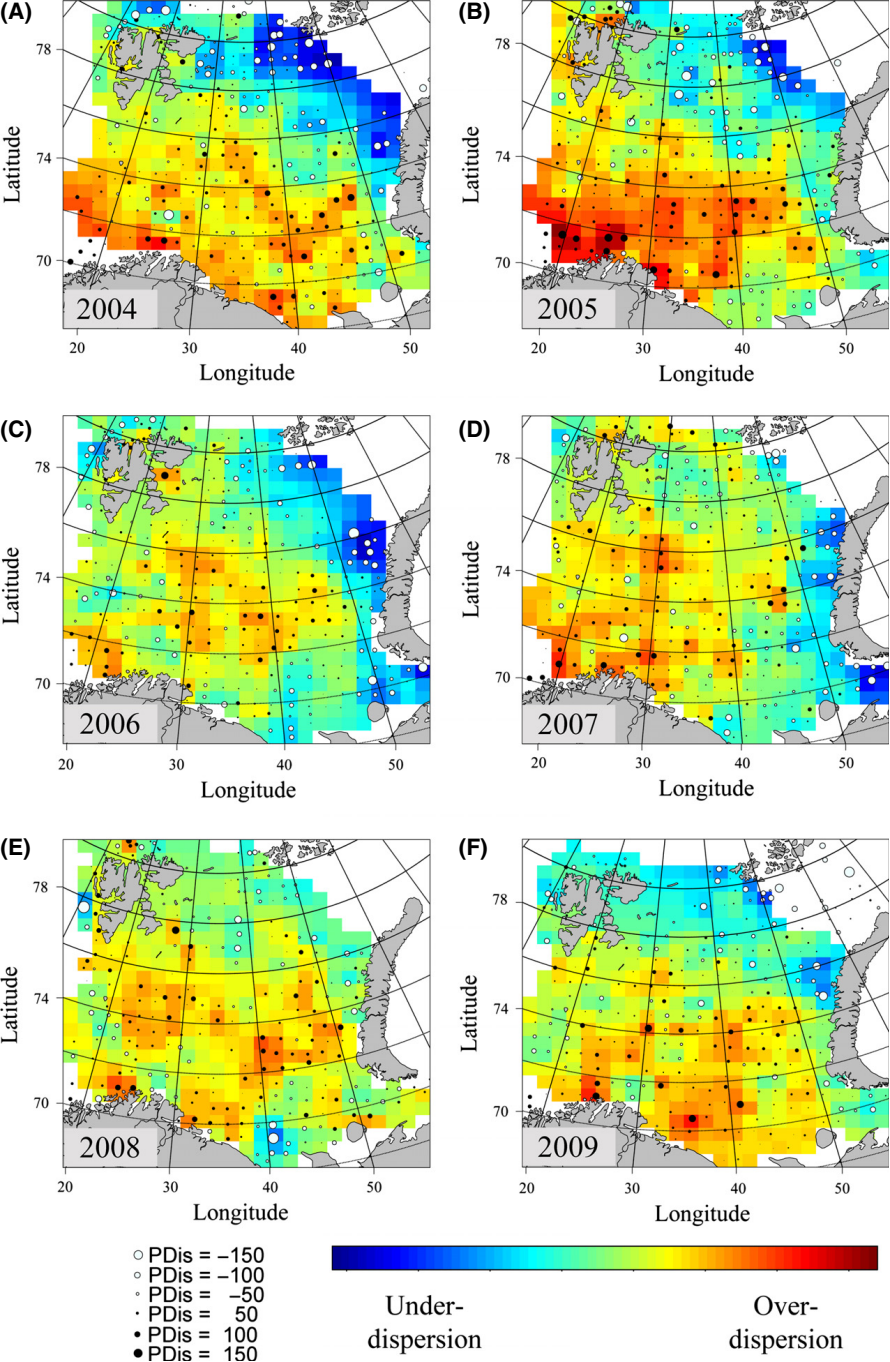
Phylogenetic dispersion of Barents Sea fish, 2004–2009. Higher values (red) indicate phylogenetic overdispersion, whereas lower values (blue) indicate phylogenetic underdispersion. (A) 2004, (B) 2005, (C) 2006, (D) 2007, (E) 2008, (F) 2009.

From the start of the study period, a marked gradient in PDis was observed, from high values (i.e., phylogenetic overdispersion) in the southwest to low values (i.e., phylogenetic underdispersion) in the northeast (Fig. 9). However, this gradient was gradually weakened in the following years. In the northeast, the increase in PDis coincided with an increase in mean FS 1 and FS 2 values.

## Discussion

### Fish life history variation

The Barents Sea fishes displayed clear patterns of life history traits covariation, with the two main life history continua capturing the fast–slow continuum and the offspring size and number continuum. The fast–slow continuum is plausibly an evolutionary effect of different selective regimes experienced in specific biogeographic areas and habitats. The offspring size and number continuum, on the other hand, is expected due to an allocation trade-off between size and number of offspring (Stearns 1992). The latter life history continuum can be detected in the data when body size is corrected for. Closely related species were similar with regard to life history characteristics, phylogeny accounting for variation in both the fast–slow and the offspring size and number continua, stressing the importance of phylogenetic constraints in life history evolution. Boreal species typically displayed slower life histories than Arctic and Arcto-boreal species, with later age and larger size at maturity, and higher longevity, the latter finding being in contrast with expectations (Liu and Walford 1966; Skúladóttir 1998; O’Brien 1999; Hansen and Aschan 2000; La Mesa and Vacchi 2001; Valenzano et al. 2006; Hildebrandt et al. 2011). Nevertheless, when correcting for maximum body size, biogeographic differences in longevity were less marked. In general, the slower species were typically boreal, demersal piscivores, with characteristics common for fishes at higher trophic levels. On the other hand, the faster species were typically pelagic planktivores.

The boreal and Arcto-boreal species had typically higher fecundity than the Arctic species, a finding which is in line with the expectation that the Arctic environment promotes larger offspring, at the cost of fecundity, to increase offspring survival. In the Arctic, where the environment is highly seasonal, the food availability for newborn larvae is very variable and can be unpredictable. Larger eggs result in larger larvae, and a large larva will become a better swimmer, with higher lipid storages and smaller relative food requirements than smaller larvae, thus being more competitive under poor feeding conditions (Marshall 1953; Duarte and Alcaraz 1989). Thus, in the Arctic, a high investment in each offspring is selectively advantageous (Marshall 1953). High fecundity is typically selected in the presence of low juvenile survival (Hutchings 2008).

### Fast–slow continua

Fish species with slow life histories are intrinsically more sensitive to exploitation than faster ones (e.g., Jennings et al. 1998). Based on ordination analyses of the life history trait matrix, species were arranged along two fast–slow gradients. Our results show that some of the most commercially important species, such as the three redfish species (*Sebastes* spp.), the Northeast Arctic cod (*Gadus morhua*), and the Atlantic halibut (*Hippoglossus hippoglossus*), appear to be particularly slow (Fig. 6). Apart from being large, which make them commercially attractive, most of these species are also long lived and late maturing. Several stocks of such species have been fished down to local extinction elsewhere (e.g., several Atlantic cod stocks in the Northwest Atlantic in the early 1990s; Bundy et al. 2009), showing a sensitivity to fishing consistent with our ranking based on life history characteristics. Interestingly, many of the Arctic fish species appear to possess faster life histories, even after correcting for maximum body size, a strategy that may constitute an adaptation to extreme and variable conditions.

### Spatial and temporal variation in life history

Our maps of average ordination ranks expand existing knowledge about the spatial heterogeneity of the Barents Sea fish community and confirm that the fish found in the south differ from those in the north with respect to life history traits (e.g., Fossheim et al. 2006; Johannesen et al. 2012a). The southwest displayed communities consisting of slow species throughout the study period, which probably reflects the boreal nature of the species found there. As such, the maps confirmed the general expectation that the smallest-sized species would be found in the northernmost, Arctic parts of the study area, whereas the larger species generally would be found in the southwest (Johannesen et al. 2012a; A. Dolgov, unpublished data). In the start of the study period, phylogenetic underdispersion in the northeast confirmed the expectation that these areas would show strong phylogenetic relatedness (Cristiansen and Reist 2013). Also, our hypothesis that the species found in the northernmost parts of the Barents Sea would have the largest offspring and the smallest fecundities (e.g., Christiansen et al. 1998) was confirmed by the present maps of average positions along the offspring size and number gradient. However, toward the later, warmer years of the study period, the fish assemblages in the northeastern corner of the Barents Sea experienced increasing phylogenetic dispersion as well as increasing FS ranks and increasing representation of fishes with smaller offspring. This probably happened as a consequence of recent northwards expansions of boreal species in the Barents Sea (Johannesen et al. 2012a), expansions that were not detected before 2004 (Aschan et al. 2013).

Fishes, which usually have rather narrow thermal windows, are often temperature sensitive and can therefore be expected to redistribute rapidly in response to climate change (Cheung et al. 2009; Last et al. 2011; Gilg et al. 2012; Poloczanska et al. 2013). Our data show that in later years, typical Arctic species (e.g., the gelatinous snailfish, *Liparis fabricii*) had to cope with a stronger representation of boreal species such as the Northeast Arctic cod (Johansen et al. 2013). In this manner, the typical Arctic species composition was apparently diluted in the warmer years, a pattern that was also clearly reflected by increasing average fast–slow values. We propose that the changing water mass characteristics associated with climate warming made such a borealization of the northern fish community possible.

### Implications for sensitivity and vulnerability to fishing

The southwest to northeast gradient in fast–slow characteristics of species may imply a higher sensitivity to fishing in the southwest. Average FS 1 ranks (which were not corrected for body size) were similar to those resulting from the intrinsic vulnerability index (Fig. S9 in appendix S3, Supporting information; Cheung et al. 2007). On the species level, our analyses confirm that both the intrinsic vulnerability index (Cheung et al. 2005) and the FS 1 ranks were strongly associated with maximum body size (Table S4 in appendix S1, Supporting information). The maximum body size is indeed an important indicator of a species’ sensitivity to exploitation (e.g., Denney et al. 2002). Nevertheless, we argue that also size-corrected fast–slow estimates, such as FS 2, provide important information about the sensitivity to fishing. At the species level, such estimates will help to identify species such as the very slow golden redfish (*Sebastes norvegicus*), a species that has a high longevity despite a relatively small body size. The golden redfish is overexploited in the Barents Sea, and currently the stock shows a major abundance decline which calls for a ban of directed fisheries until major improvement is evident (ICES 2013). Integrated at the fish assemblage level, the size-corrected FS 2 values also displayed clear signs of a borealization in the northeast leading to assemblages that are more sensitive to fishing. Assuming that global temperatures will continue to increase, particularly in high-latitude areas (ACIA 2004; Smedsrud et al. 2013), we support the expectation that northward movements of boreal, commercially attractive species will be more prominent in coming years (Drinkwater 2005; Stenevik and Sundby 2007). As a consequence, the northernmost regions in the Barents Sea will likely become more interesting to the fishing industry (Christiansen et al. 2014).

### Fish vulnerability and fishery management

We argue that fast–slow continua, due to their demographic implications, are important indicators for the species’ ability to withstand systematic exploitation. Defining vulnerability as the extent to which experienced stress (i.e., exploitation) may harm the species (Reynolds et al. 2008), we therefore suggest that FS continua constitute important components of a fish species’ vulnerability to fishing. Such information may be taken into account, for instance in the Barents Sea Management Plan (Olsen et al. 2007). Managers should also account for variation in catchability, escape from fishing gear, and postselection mortality of nontarget species (Suuronen 2005).

At present, fishing occurs in the southern and northwestern parts of the Barents Sea, where the overall sensitivity of the fish community is relatively high (ICES 2013). This suggests that the fishes in the southwestern Barents Sea may be vulnerable and should be managed with caution. As discussed previously, increasing fishing pressure can be expected in the north in the near future due to a rising representation of commercially attractive species. This highlights the need for cautious and knowledge-based management, which can be supported by further work on trait-based methods. However, a continuous monitoring of species’ life history traits is necessary, not only due to the fact that selective fishing induces rapid evolution (Jørgensen et al. 2007), but also because rapid changes in life histories may provide early warnings about serious population declines (Olsen et al. 2004). In this respect, we encourage future research programmes to expand the knowledge of fish life history traits for specific areas such as the Barents Sea, in order to enable more precise analyses of the species’ sensitivity to fishing. Although this study suggests that fish species found in the northeast exhibit life history traits that may make them comparatively less sensitive to fishing, commercial fishing in the northeast should be carefully planned due to the multiple stressors occurring there and due to the limited knowledge of the biology in the area (Christiansen et al. 2014).

## Supporting Information

Appendix S1 Supplementary tables, including life history trait matrix, citations for the life history trait matrix, sampling locations of trait data, correlation table, explanatory variables and a nested taxonomic matrix for Barents Sea fish.

**Appendix S2.** Reference list for the life history trait matrix. **Appendix S3.** Supplementary figures.
