# Supplementary material for: Life history variation in Barents Sea fish: implications for sensitivity to fishing in a changing environment
Source: Ecol Evol. 2014 Sep 2;4(18):3596–611. doi: 10.1002/ece3.1203 (PMC4224534; doi:10.1002/ece3.1203)
Supplement: Supplementary file 2 — Appendix S2. Reference list for the life history trait matrix. Appendix S3. Supplementary figures. [file ece30004-3596-sd2.docx]

Supporting Information

for:

**Life history variation in Barents Sea fish: implications for sensitivity to fishing in a changing environment**

Magnus Aune Wiedmann, Raul Primicerio, Andrey Dolgov^­^, Camilla A. Meyer Ottesen, and Michaela Aschan

**Appendix S2: References and notes associated with the life history trait matrix.**

Andriyashev, A.P. (1954) *Fishes of northern seas of the U.S.S.R.* Academy of Science Press, Moscow-Leningrad. Translated from Russian by the Israel Program for Scientific Translations, Jerusalem 1964.

Bagenal, T.B. (1965) The fecundity of witches in the Firth of Clyde. *Journal of the Marine Biological Association of the United Kingdom,* **43,** 401-407.

Bergstad, O.A., Jørgensen, T. & Dragesund, O. (1987) Life history and ecology of the gadoid resources of the Barents Sea. *Fisheries Research,* **5,** 119-161.

Burnett, J., Ross, M.R. & Clark, S.H. (1992) Several biological aspects of the witch flounder (*Glyptocephalus cynoglossus* (L.)) in the Gulf of Maine- Georges Bank region. *Journal of Northwest Atlantic Fishery Science,* **12,** 15-25.

Calis E, Jackson EH, Nolan CP, Jeal F (2005) Preliminary age and growth estimates of the rabbitfish, *Chimara monstrosa*, with implications for future resource management. *Journal of Northwest Atlantic Fishery Science,* **35**, 15-26.

Casey, J.M. & Myers, R.A. (1998) Near extinction of a large, widely distributed fish. *Science,* **281,** 690-692.

Christiansen, S.J., Fevolden, S.E., Karamuschko, O.V. & Karamuschko, L.I. (1998) Maternal output in polar fish reproduction. *Fishes of Antarctica. A biological overview* (eds G. Di Prisco, E. Pisano & A. Clarke), pp. 41-51, Springer Verlag, Milano, Italy.

Clarke, M.W., Kelly, C.J., Connolly, P.L. & Molloy, J.P. (2003) A life history approach to the assessment and management of deep-water fisheries in the Northeast Atlantic. *Journal of Northwest Atlantic Fishery Science,* **31,** 401-411.

Coad, B.W. & Power, G. (1973) Observations on the ecology and phenotypic variation of the threespine stickleback, *Gasterosteus aculeatus* L., 1758, and the blackspotted stickleback, *G. wheatlandi* Putnam, 1867 (Osteichthyes: Gasterosteidae) in Amory Cove, Quebec. *The Canadian Field-Naturalist,* **87,** 113-122.

Dolgov, A.V. (2009) Trophic structure of the Barents Sea fish assemblage with special reference to the cod stock recoverability. *Progress in Oceanography,* **81,** 165-173.

Dolgov, A.V., Mukhina, N.V., Dolgova, N.V. & Yaragina, N.A. (2004) Otoliths of marine fish of the Barents Sea. Third international symposium on fish otolith research and application, 11-16 July 2004. Handbook and abstracts, p. 114.

Drevetnyak, K.V. & Kluev, A.I. (2005) On fecundity of *Sebastes viviparus* from the North East Arctic. *ICES CM 2005,* **Q:31**.

Eliassen, J.E. & Falk-Petersen, I.B. (1985) Reproductive biology of the roughhead grenadier (*Macrourus berglax* Lacepède) (Pisces, Gadiformes) from the continental slope of Northern Norway. *Sarsia*, **70,** 59-67.

Erzini, K. (1994) An empirical study of variability in length-at-age of marine fishes. *Journal of Applied Ichthyology,* **10,** 17-41.

Falk-Petersen, I.B., Frivoll, V., Gulliksen, B., Haug, T. & Vader, W. (1988) Age/size relations and food of two snailfishes, *Liparis gibbus* and *Careproctus reinhardii* (Teleostei, Liparididae) from Spitsbergen coastal waters. *Polar Biology,* **8,** 353-358.

Frisk, M.G., Miller, T.J. & Fogarty, M.J. (2001) Estimation and analysis of biological parameters in elasmobranch species: a comparative life history study. *Canadian Journal of Fisheries and Aquatic Sciences,* **58,** 969-981.

Froese, R. & Pauly, D. (2013) Fishbase (http://www.fishbase.com, accessed: 19 November 2013).

Gjøsæter, H. (1998) The population biology and exploitation of capelin (*Mallotus villosus*) in the Barents Sea. *Sarsia,* **83,** 453-496.

Gjøsæter, J. (1973) Preliminary results of Norwegian polar cod investigation 1970-1972. *ICES CM 1973,* **F:8**.

Gjøsæter, J. (1981a) Life history and ecology of *Maurolicus muelleri* (Gonostomatidae) in Norwegian waters. *Fiskeridirektoratets Skrifter Serie Havundersøkelser,* **17,** 109-131.

Gjøsæter, J. (1981b) Growth, production and reproduction of the myctophid fish *Benthosema glaciale* from western Norway and adjacent seas. *Fiskeridirektoratets Skrifter Serie Havundersøkelser,* **17,** 79-108.

Gundersen, A.C., Kjesbu, O.S., Nedreaas, K.H. & Stene, A. (1999) Fecundity of Northeast Arctic Greenland halibut (*Reinhardtius hippoglossoides*). *Journal of Northwest Atlantic Fishery Science,* **25,** 29-36.

Haug, T. & Gulliksen, B. (1988) Fecundity and oocyte sizes in ovaries of female Atlantic halibut. *Sarsia,* **73,** 259-261.

Hildebrandt, N., Bergmann, M. & Knust, R. (2011) Longevity and growth efficiency of two deep-dwelling Arctic zoarcids and comparison with eight other zoarcid species from different climatic regions. *Polar Biology,* DOI: 10.1007/s00300-011-1011-4.

Huse, G. & Gjøsæter, H. (1997) Fecundity of the Barents Sea capelin (*Mallotus villosus*). *Marine Biology,* **130,** 309-313.

Il’inskiy, E.N., Balanov, A.A. & Ivanov, O.A. (1995) Rare species- *Scopelosaurus harryi*, *Arctozenus rissoi*, *Magnisudis atlantica*, and *Tactostoma macropus* from the Northwest Pacific. 2. Spatial distribution and biology. *Journal of Ichthyology,* **35,** 1-19.

Jákupsstovu, S.H.I. & Haug, T. (1988) Growth, sexual maturation, and spawning season of Atlantic halibut, Hippoglossus hippoglossus, in Faroese waters. *Fisheries Research,* **6,** 201-215.

Jennings, S., Reynolds, J.D. & Mills, S.C. (1998) Life history correlates of responses to fisheries exploitation. *Proceedings of the Royal Society of London B: Biological Sciences,* **265,** 333-339.

Jennings, S., Greenstreet, S.P.R. & Reynolds, J.D. (1999) Structural change in an exploited fish community: a consequence of differential fishing effects on species with contrasting life histories. *Journal of Animal Ecology,* **68,** 617-627.

Magnussen, E. (2007) Interpopulation comparison of growth patterns of 14 fish species on Faroe Bank: are all fishes on the bank fast-growing? *Journal of Fish Biology,* **71,** 453-475.

Morua, H. (2003) Population structure, growth and reproduction of roughhead grenadier on the Flemish Cap and Flemish Pass. *Journal of Fish Biology,* **63,** 356-373.

Moura, T., Figueiredo, I., Machado, P.B. & Gordo, L.S. (2004) Growth pattern and reproductive strategy of the holocephalan *Chimaera monstrosa* along the Portuguese continental slope. *Journal of the Marine Biological Association of the United Kingdom,* **84,** 801-804.

Munk, P. & Nielsen, J.G. (2005) *Eggs and larvae of North Sea fishes.* biofolia, Fredriksberg, Denmark.

Møller, P.R. & Jørgensen, O.A. (2000) Distribution and abundance of eelpouts (Pisces, Zoarcidae) off West Greenland. *Sarsia,* **85,** 23-48.

Nash, R.D.M. (1986) Aspects of the general biology of Wahl’s eelpout, *Lycodes vahlii gracilis* M. Sars, 1867 (Pisces: Zoarcidae) in Oslofjorden, Norway. *Sarsia,* **71,** 289-296.

Newton, A.W. & Armstrong, D.W. (1974) A note on the fecundity of lemon sole. *ICES CM 1974,* **F:34**.

Ni, I.H. & Sandeman, E.J. (1984) Size at maturity for Northwest Atlantic redfishes (*Sebastes*). *Canadian Journal of Fisheries and Aquatic Sciences,* **41,** 1753-1762.

Oldham, W.S. (1972) Biology of the Scotian Shelf cusk, *Brosme brosme*. *International Commission for the Northwest Atlantic Fisheries Research Bulletin,* **9,** 85-98.

Pauly, D. (1978) A preliminary compilation of fish length growth parameters. *Berichte des Institut für Meereskunde an der Universität Kiel,* **55**.

Penney, R. (1987) Development of deep-water redfish (*Sebastes mentella*) larvae, with comparative notes on newly extruded Acadian redfish (*Sebastes fasciatus*) from the Flemish Cap. *Canadian Journal of Zoology,* **65,** 1167-1180.

Pethon, P. (2005) *Aschehougs store fiskebok.* Aschehoug, Oslo, Norway (in Norwegian).

Raitt, D.F.S. & Hall, W.B. (1967) On the fecundity of the redfish. *Journal du Conseil/ Conseil Permanent International pour l’Exploration de la Mer,* **31,** 237-245.

Rasmussen, O.I. & Giske, J. (1994) Life-history parameters and vertical distribution of *Maurolicus muelleri* in Masfjorden in summer. *Marine Biology,* **120,** 649-664.

Salvanes, A.G.V. & Stockley, B.M. (1996) Spatial variation of growth and gonadal developments of *Maurolicus muelleri* in the Norwegian Sea and in a Norwegian fjord. *Marine Biology,* **126,** 321-332.

Snyder, R.J. (1991) Migration and life histories of the threespine stickleback: evidence for adaptive variation in growth rate between populations. *Environmental Biology of Fishes,* **31,** 381-388.

Sulikowski, J.A., Kneebone, J., Elzey, S., Jurek, J., Danley, P.D., Howell, W.H. & Tsang, P.C.W. (2005) Age and growth estimates of the thorny skate (*Amblyraja radiata*) in the western Gulf of Maine. *Fishery Bulletin,* **103,** 161-168.

Tokranov, A.M. & Orlov, A.M. (2005) Some features of the biology of *Icelus spatula* (Cottidae) in Pacific waters off the northern Kuril Islands. *Journal of Ichthyology,* **45,** 229-236.

Tåning, Å.V. (1961) Larval and postlarval stages of *Sebastes* species and *Helicolenus dactylopterus*. *Rapports et procès-verbaux des réunions / Conseil permanent international pour l'exploration de la mer,* **150,** 234-240.

Walker, P.A. & Hislop, J.R.G. (1998) Sensitive skates or resilient rays? Spatial and temporal shifts in ray species composition in the central and north-western North Sea between 1930 and the present day. *ICES Journal of Marine Science,* **55,** 392-402.

Walsh, S.J. (1994) Life history traits and spawning characteristics in populations of long rough dab (American plaice) *Hippoglossoides platessoides* (Fabrisius) in the North Atlantic. *Netherlands Journal of Sea Research,* **32,** 241-254.

Website: <http://www.elasmo-research.org/education/ecology/polar-greenland.htm>. Accessed December 15 2013.

Wienerroither, R., Johannesen, E., Dolgov, A., Byrkjedal, I., Bjelland, O., Drevetnyak, K., Eriksen, K.B., Høynes, Å., Langhelle, G., Langøy, H., Prokhorova, T., Prozorkevich, D. & Wenneck, T. (2011) Atlas of the Barents Sea fishes. *IMR/PINRO Joint Report Series,* **1,** 1-272.

**Appendix S3: Supplementary figures**


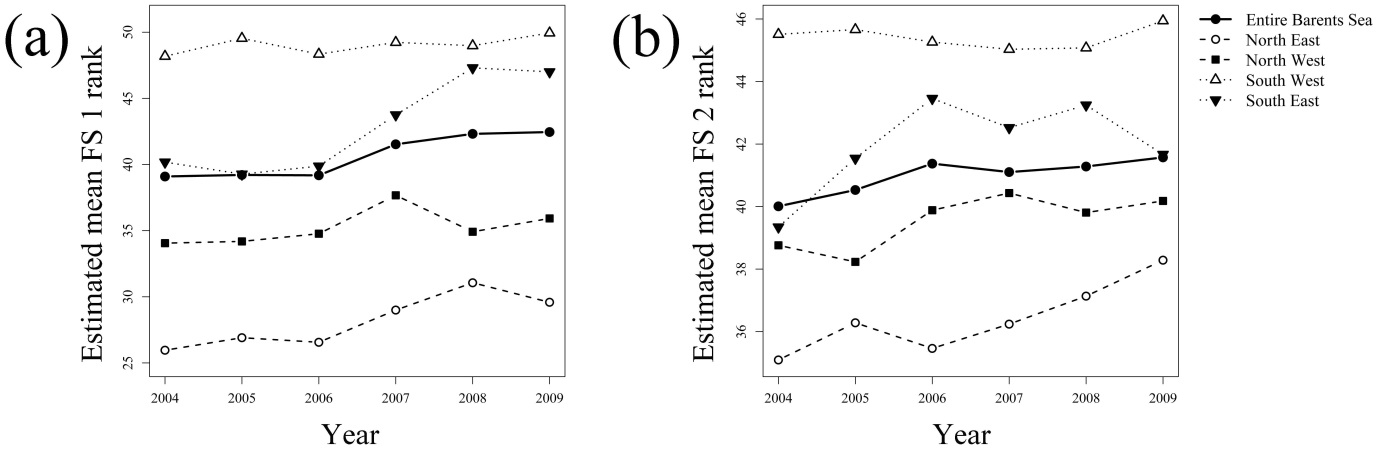


**Fig. S7.** Estimated mean FS ranks of Barents Sea fish during the years 2004-2009, for the whole Barents Sea area and for the four corners of the area separately. To study these four corners of the Barents Sea separately, we identified the spatial midpoint of the survey stations (75.1°N, 38.1°E) and divided the Barents Sea into 4 equal-sized regions. Applying the GLM that was used on the full Barents Sea dataset, we modelled the average FS 1 (a) and FS 2 (b) ranks in these four regions separately.


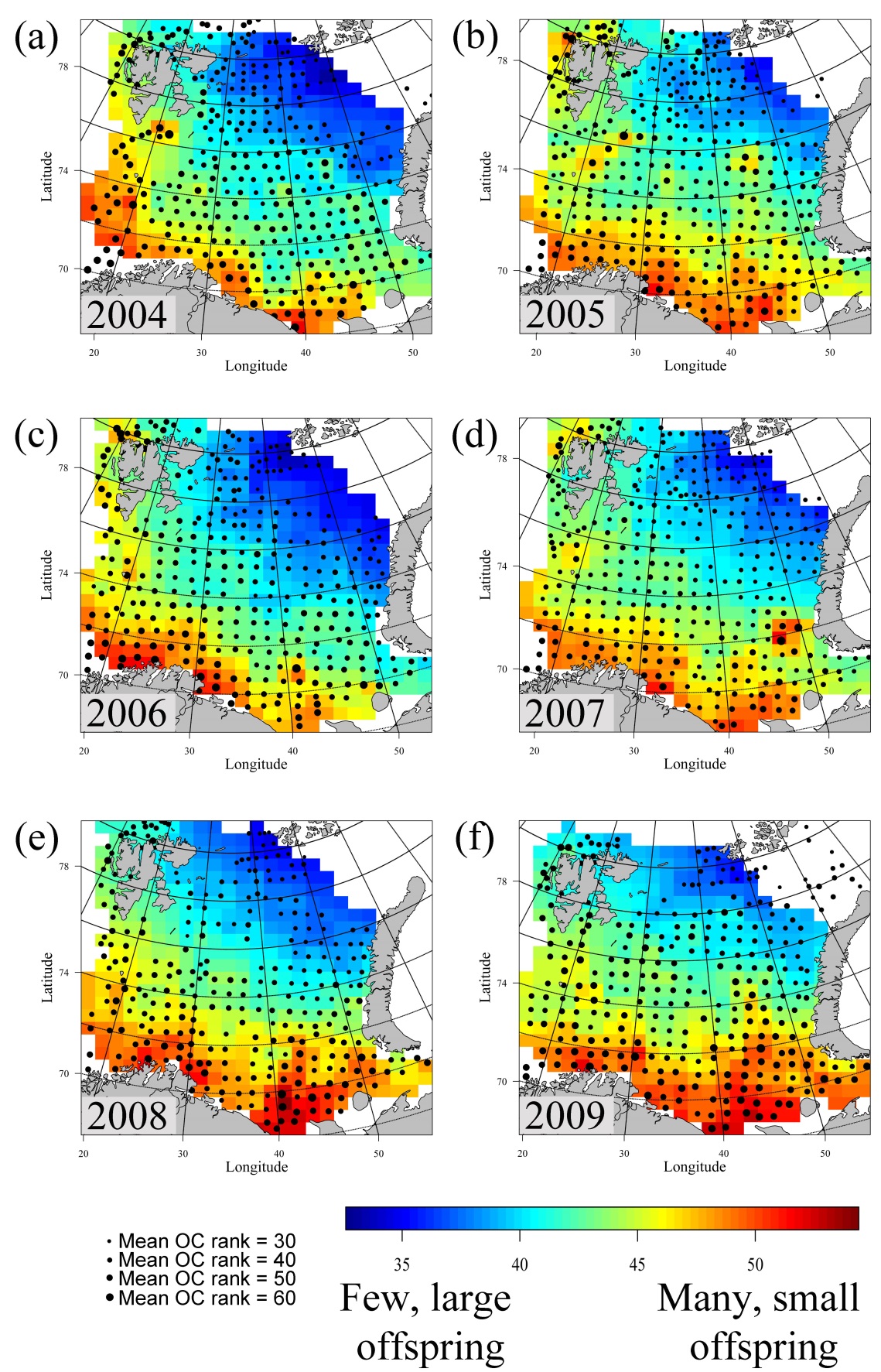


**Fig. S8.** Mean rank values of the offspring size and number gradient. High values (red colours) reflect fish with many, small offspring, whereas low values (blue colours) reflect fish with few, large offspring. (a) 2004, (b) 2005, (c) 2006, (d) 2007, (e) 2008, (f) 2009.


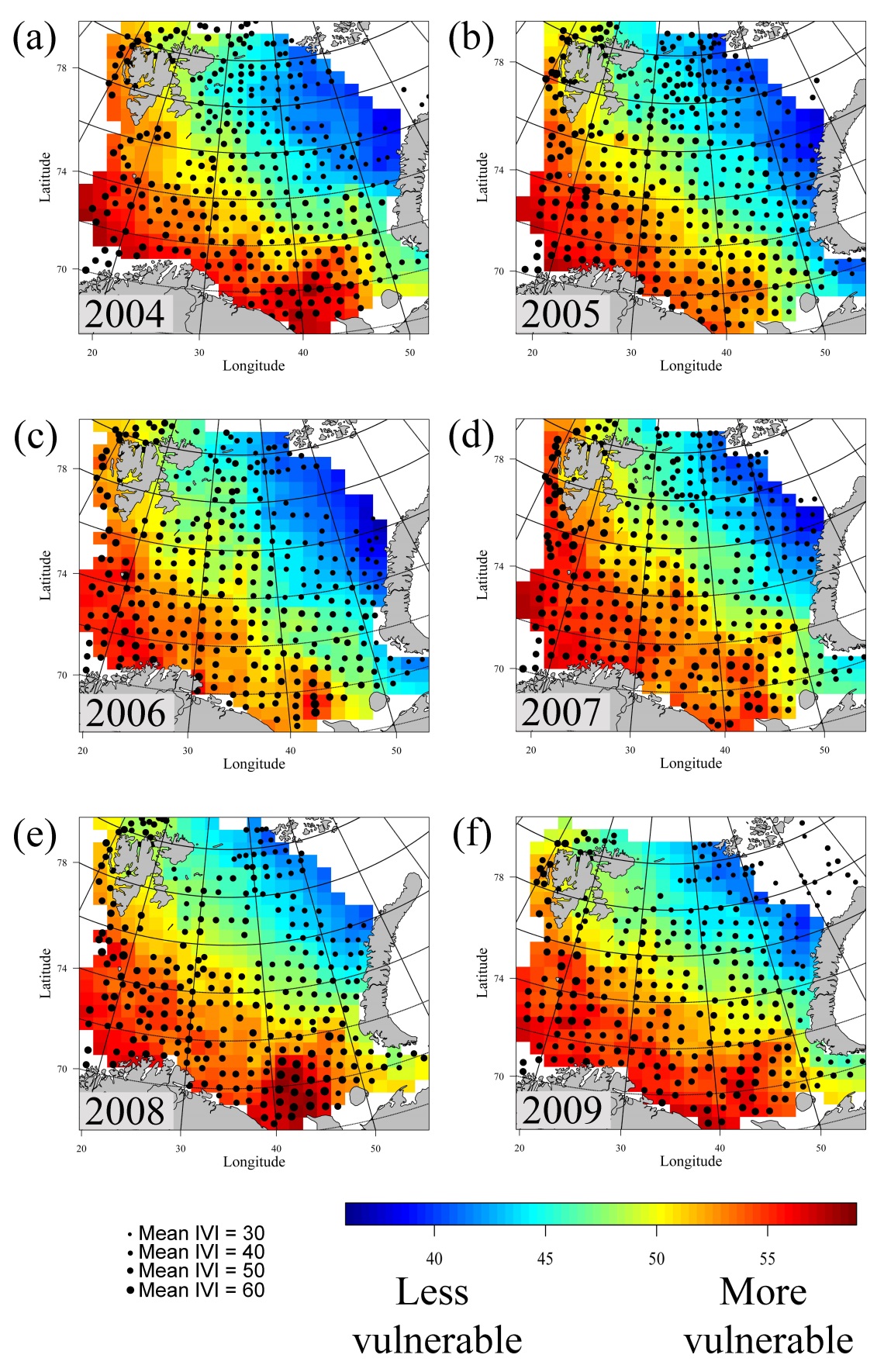


**Fig. S9.** Mean community-level vulnerability of fish based on the intrinsic vulnerability indices (IVI) available from www.fishbase.org (Cheung, Pitcher & Pauly 2005; Froese & Pauly 2013). (a) 2004, (b) 2005, (c) 2006, (d) 2007, (e) 2008, (f) 2009.

**References**

Cheung, W.W.L., Pitcher, T. & Pauly, D. (2005) A fuzzy logic expert system to estimate intrinsic extinction vulnerabilities of marine fishes to fishing. *Biological Conservation,* **124,** 97-111.

Froese, R. & Pauly, D. (2013) Fishbase (http://www.fishbase.com, accessed: 19 November 2013).
